# Development of a DNA isolation device using poly(3,4-dihydroxy-L-phenylalanine)-coated swab for on-site molecular diagnostics

**DOI:** 10.1038/s41598-019-44527-2

**Published:** 2019-05-31

**Authors:** Hyun-Ju Park, Heesoo Cho, Ho Sang Jung, Baek Hwan Cho, Min-Young Lee

**Affiliations:** 1 Department of Medical Device Management and Research, Samsung Advanced Institute for Health Sciences & Technology (SAIHST), Sungkyunkwan University, Seoul, 06351 Korea; 2 Smart Healthcare Research Institute, Biomedical Engineering Research Center, Samsung Medical Center, 81, Irwon-ro, Gangnam-gu, Seoul, 06351 Korea; 3 Advanced Nano-Surface Department, Korea Institute of Materials Science (KIMS), Changwon, Gyeongnam, 51508 Republic of Korea

**Keywords:** Lab-on-a-chip, Genetic testing

## Abstract

For on-site molecular diagnostics, a pre-treatment step for isolation of nucleic acid from clinical samples on site is desired. However, conventional commercialized silica-based nucleic acid isolation kits require repetitive pipetting and a centrifugation or permanent magnet for buffer exchange. In this study, we developed a poly(3,4-dihydroxy-L-phenylalanine) (L-DOPA)-coated swab that can absorb and desorb DNA depending on pH of buffers and a portable integrated DNA isolation device that comprises integrated chambers containing DNA isolation buffers. The poly(L-DOPA)-coated swab interacts with each buffer by passing through the membrane between the integrated chambers. Our device involves a simple operation and does not require any large equipment or skilled experts. By connecting the device with an automated polymerase chain reaction system, an isothermal amplification system, or a non-amplified DNA detection method, on-site molecular diagnosis of various diseases can be quickly realized.

## Introduction

On-site molecular diagnostics can help prevent transmission of infectious diseases and monitor the safety of foods and water supplies^1^. The demand for on-site molecular diagnostics for early diagnosis and healthcare has seen a rapid increase. The objective of on-site molecular diagnostics is to make it almost as easy-to-use as a lateral flow strip test device such as a pregnancy test kit or an electro-enzymatic sensor such as a blood glucose sensor^2^. Recently, a variety of nucleic acid (NA) analysis technologies for point-of-care testing (POCT) have been developed, including NA isothermal amplification methods (loop-mediated isothermal amplification^3^, rolling circle amplification^4^, and nicking enzyme amplification reaction^5^ etc.), LED or colorimetric-based amplified NA detection methods^6,7^ and non-amplified NA detection methods using piezoelectric^8^ or gold nanoparticles^9^. However, patient specimens include inhibitors that interfere with NA amplification and detection strategies. Nucleic acid isolation (NAI) is one of the pivotal steps as a starting point for molecular diagnostics. To realize on-site molecular diagnosis, pre-treatment of clinical samples, which includes high-quality NAI, should be performed on site^10^. This important procedure has substantially evolved over the past several decades; however, some more research is required before the NAI protocol used in laboratory environments can be used in POCT environments as well. Conventional commercialized NAI kits use silica gel filters or silica-coated magnetic beads^11^. These kits are designed to perform the process in a single tube, so repetitive pipetting and a centrifugation or permanent magnet are required to exchange buffers for lysis, binding, washing, and elution^11^. Although automated DNA isolation equipment have been developed that automatically exchange buffers, they are large and expensive for handling large samples in large hospitals^12^. Hali Bordelon *et al*. reported an NAI method that does not require exchange of the buffers, wherein the buffers are separated by small air gaps in a small diameter tube and silica-coated magnetic beads interact with each buffer while moving the external permanent magnet^13^. However, separation by small air gaps between buffers has instability, so that the buffers can be mixed during the dealing process. Microfluidic devices for automated NAI have been developed, but most of these devices are able to process only small sample volumes (usually < 100 μL) and require pumping equipment for flow control^14^.

In this study, we developed a portable integrated DNA isolation device that does not require complex buffer exchange process and large equipment for on-site molecular diagnostics. This device comprises a connected chamber containing DNA isolation buffers and a poly(3,4-dihydroxy-L-phenylalanine) (L-DOPA)-coated swab. It can be operated simply by passing the swab through the membrane between the chambers. It is convenient and can be safely used without the need for any skilled expert to be present. We evaluated the DNA isolation efficiency of the poly(L-DOPA)-coated swab compared with commercialized NAI kits. Furthermore, we confirmed that the DNA isolated from the cell in the integrated device is capable of NA amplification.

## Results and Discussion

### Strategy of DNA isolation using integrated DNA isolation device

**Figure 1:**
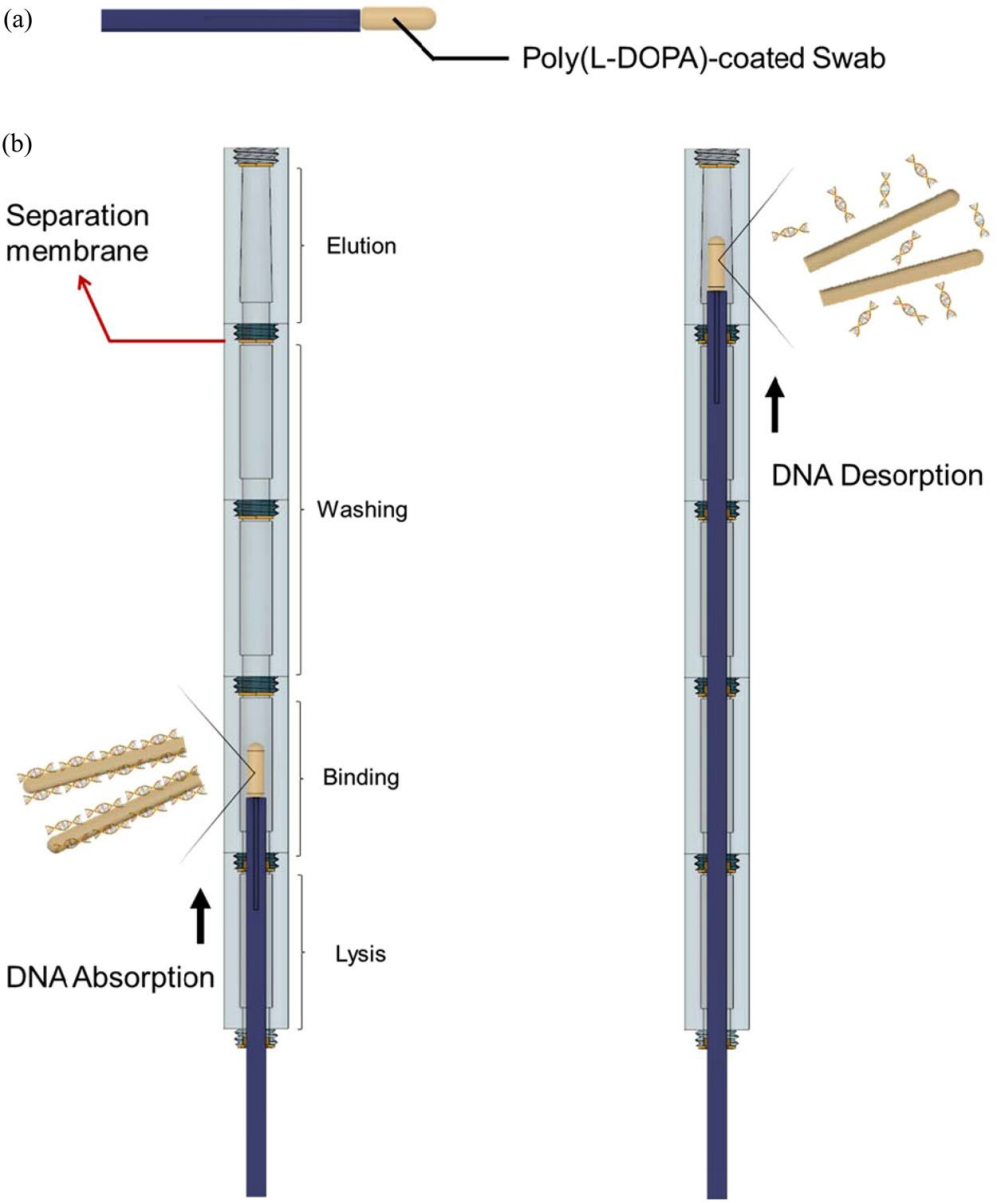
Overview of integrated DNA isolation device. (**a**) Poly(L-DOPA)-coated swab and (**b**) integrated DNA isolation device comprises a series of chambers containing lysis, binding, washing, and elusion buffers.

We devised an integrated DNA isolation device to easily isolate DNA from samples without large external equipment or pipetting. As shown in Fig. 1, the integrated DNA isolation device comprises a series of chambers containing lysis, binding, washing, and elusion buffers and each chamber is separated by a membrane. The poly(L-DOPA)-coated swab can interact with each buffer inside the chambers by passing through the membrane. This device does not require a pipette, a centrifuge, and an external permanent magnet for buffer exchange.

The operation protocol of the integrated DNA isolation device is as follows. First, we swab the specimens using the poly(L-DOPA)-coated swab. Then, we push the swab into the lysis chamber. After lysis, we move the swab to the next chamber and let it interact with the binding, washing, and elution buffers. Subsequently, we collect the isolated DNA solution from the last membrane. The isolated DNA can then be used for NA analysis such as in polymer chain reaction (PCR), isothermal amplification reaction, or non-amplification detection methods.

### Preparation of the poly(L-DOPA)-coated swab

**Figure 2:**
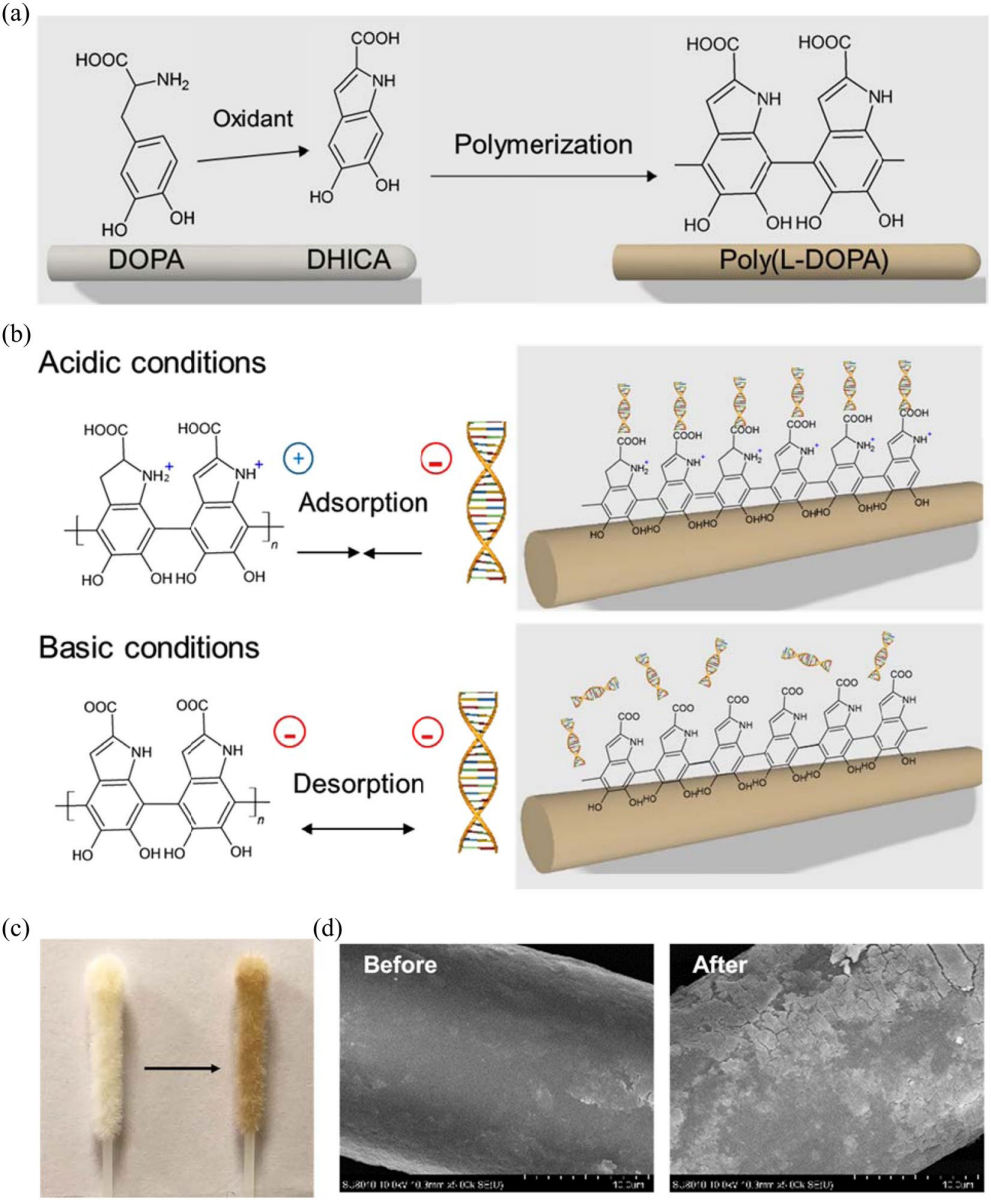
Property of poly(L-DOPA) coated on swab. (**a**) Mechanism of poly(L-DOPA) synthesis. (**b**) Mechanism of poly(L-DOPA) as a pH-responsive platform for capture/release DNA. (**c**) Photograph of swab before (left) and after (right) modified with poly(L-DOPA). (**d**) Scanning electron microscope images of swab before and after modification with poly(L-DOPA).

The swab (Copan Diagnostics, CA, USA) used in this study is commercially available for collecting nasopharyngeal specimens to diagnose respiratory infections. This swab is made of nylon. Silica, which is commonly used as a DNA isolation material, is difficult to coat on fiber materials such as nylon. Poly(dopamine) has been reported to have the ability to absorb and desorb DNA depending on pH condition^15^. However, the poly(dopamine) synthesized from dopamine exhibits a strong noncovalent bonding force such as pi stacking and hydrophobic interaction; owing to this, non-covalently bonded free poly(dopamine) and dopamine monomers can be released from the coating film in acidic or basic solutions. Through the UV–vis absorption spectra, we could observe that uncoated free poly(dopamine) was continuously released from the poly(dopamine)-coated swab in DNA isolation buffers even after several washes. This phenomenon can interfere with the NA amplification reactions or nucleic acid detection strategies. In contrast, poly(L-DOPA) using L-DOPA as a precursor was reported to have less noncovalent binding and more stable coatings on nylon than poly(dopamine)^15^. Therefore, we chose poly(L-DOPA) as a coating material on swabs for DNA isolation. We immersed the bare swab in the L-DOPA solution (2 mg/mL, pH 8.5) for 20 h to undergo a reaction. It oxidized to form 5,6-dihydroxyindole-2-carboxylic acid (DHICA), which is polymerized into poly(L-DOPA) with brown color and considerable stickiness (Fig. 2a). We improved its stability through oxidation and neutralization of the poly(L-DOPA)-coated swab. The oxidation process enhances the bond strengths in the poly(L-DOPA) film and between the swab and the poly(L-DOPA)^16^. We washed the poly(L-DOPA)-coated swab using acidic (pH 5) and alkaline (pH 8.5) solutions. This procedure was repeated until no free poly(L-DOPA) or L-DOPA was observed in the UV–vis spectra of the wash solutions. As shown in Fig. 2b, poly(L-DOPA) contains carboxyl groups and amine groups together and is hence positively charged at acidic conditions and negatively charged at basic conditions. Using these properties, it was expected that negatively charged DNA could be adsorbed and desorbed on the poly(L-DOPA)-coated swab according to the pH condition of the buffers. The color of the swab changes from pale yellow to dark brown after coating with poly(L-DOPA) and is light brown after the oxidation, neutralization, and washing processes (Fig. 2c). The scanning electron microscope images taken before and after coating steps showed that the swab surface was coated with a poly(L-DOPA) film (Fig. 2d).

### DNA isolation using the poly(L-DOPA)-coated swab

**Figure 3:**
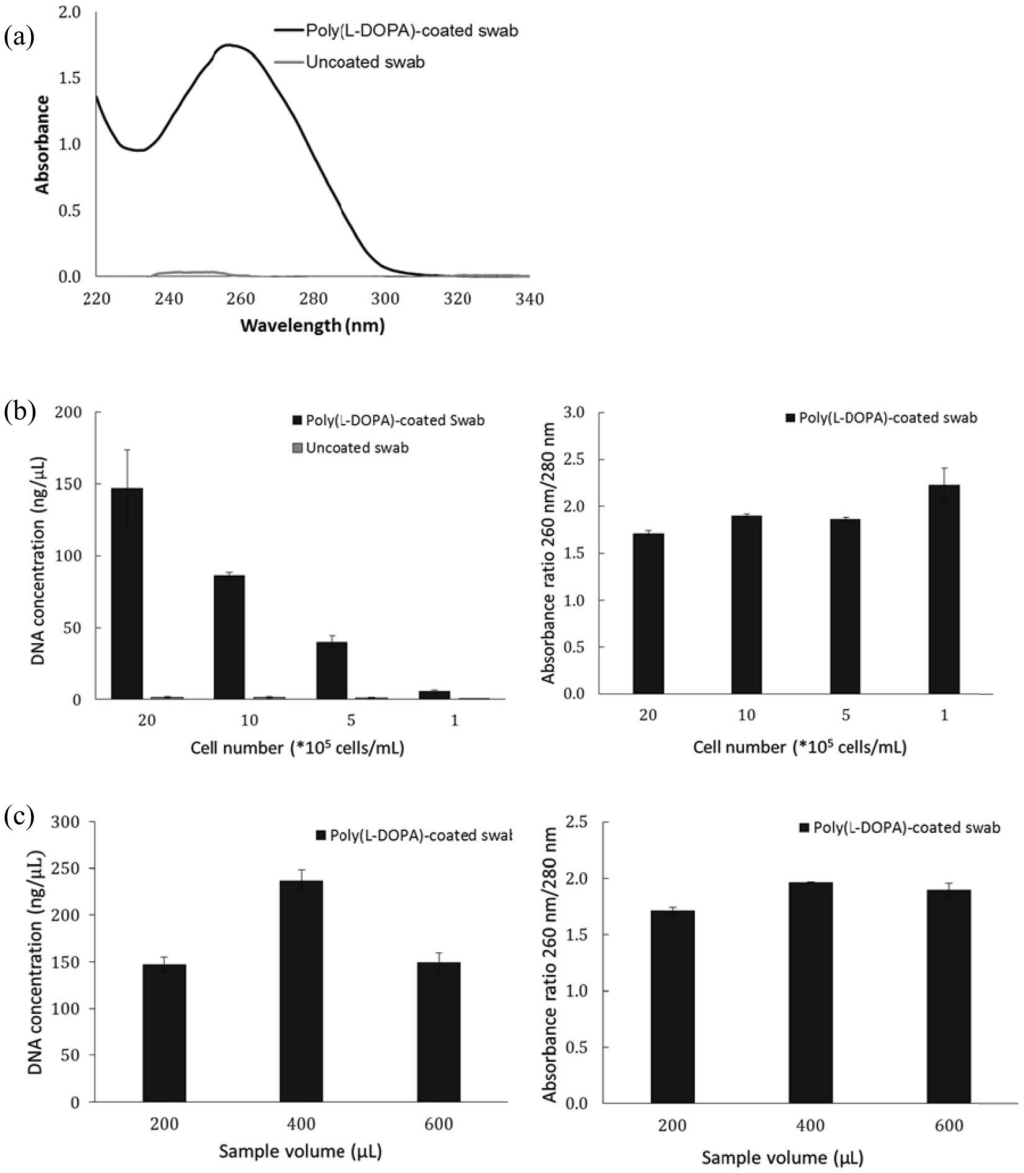
DNA isolation using poly(L-DOPA)-coated swab. (**a**) UV-vis absorbance spectra of isolated DNA using poly(L-DOPA)-coated swab and uncoated swab. The isolated DNA amounts (left) and its absorbance ratios of 260 nm/280 nm (right) according to (**b**) initial cell number and (**c**) initial sample volume.

DNA isolation using the poly(L-DOPA)-coated swab was performed depending on the cell concentration using PC-3M cells. If the pH of the binding buffer is too low, the DNA can be damaged, and if the pH of the elution buffer is too high, it can affect the NA analysis. So, in this study, the optimized DNA isolation buffers in MagListo™ 5 M Genomic DNA Extraction Kit (Bioneer, Korea Daegeon) were used. The pH of the binding buffer was approximately 5.8 and that of the elution buffer was approximately 8.53. PC-3M cells were added to the lysis buffer to obtain a cell number of 2 × 10^6^, 1 × 10^6^, 5 × 10^5^, and 1 × 10^5^. To confirm the effect of poly(L-DOPA), uncoated swab was used as a control. The UV–vis spectra of the elution buffer after DNA isolation using the poly(L-DOPA)-coated swab was shown in Fig. 3a. The absorption peak was at approximately 260 nm, which is the wavelength of maximum absorption of DNA. The isolated DNA concentration by the poly(L-DOPA)-coated swab from the PC-3M cells was 144.23 ng/µL for 2 × 10^6^ cells, 86.50 ng/µL for 1 × 10^6^ cells, 40.27 ng/µL for 5 × 10^5^ cells, and 5.93 ng/µL for 1 × 10^5^ cells. As the number of cells increased, the concentration of isolated DNA increased. In contrast, almost no DNA was isolated from uncoated swab. This result confirmed that DNA was isolated by the poly(L-DOPA) coating. The absorbance ratios of 260 nm/280 nm were observed to be over 1.7, which indicates that the isolated DNA was of high purity.

Swabs are not only able to collect sample by scraping, but they can also collect substances from a large volume of liquid sample (eg, urine) by shaking. We evaluated the DNA isolation efficiency of the poly(L-DOPA)-coated swab depending on the sample volume. The initial volumes of the lysis buffer were 200, 400, and 600 μL. The PC-3M cells were added to the buffer at a constant cell number of 2 × 10^6^ cells. The binding buffer volume was increased in proportion to the lysis buffer. The isolated DNA concentration by poly(L-DOPA)-coated swab were 147.23 ng/µL from 200 μL, 237.13 ng/µL from 400 μL, and 149.23 ng/µL from 600 μL. The absorbance ratios of 260 nm/280 nm were 1.7, 2.0, and 1.9, respectively. The poly(L-DOPA)-coated swab was observed to have the highest isolation efficiency when the sample volume was 400 μL. By increasing the size of the swap, the liquid sample volume that can be processed will increase further.

### DNA isolation efficiency compared with commercialized NAI kits

We compared the DNA isolation efficiency of the poly(L-DOPA)-coated swab with the following commercial DNA isolation kits—magnetic bead-based MagListo™ 5 M Genomic DNA Extraction Kit (Bioneer, Korea Daegeon) and silica gel filter-based DNeasy Blood & Tissue Kit (Qiagen, German Hilden). In comparison with each commercialized kit, the buffers, its volumes, and incubation times of each kit were also applied to the poly(L-DOPA)-coated swab. In the DNeasy Blood & Tissue Kit, the binding buffer pH was approximately 5.8 and the elution buffer pH was approximately 8.8, which were not significantly different from that observed for the MagListo™ 5 M Genomic DNA Extraction Kit. The final elution buffer volume was set to 300 µL to allow the swab to fully submerge.

**Figure 4:**
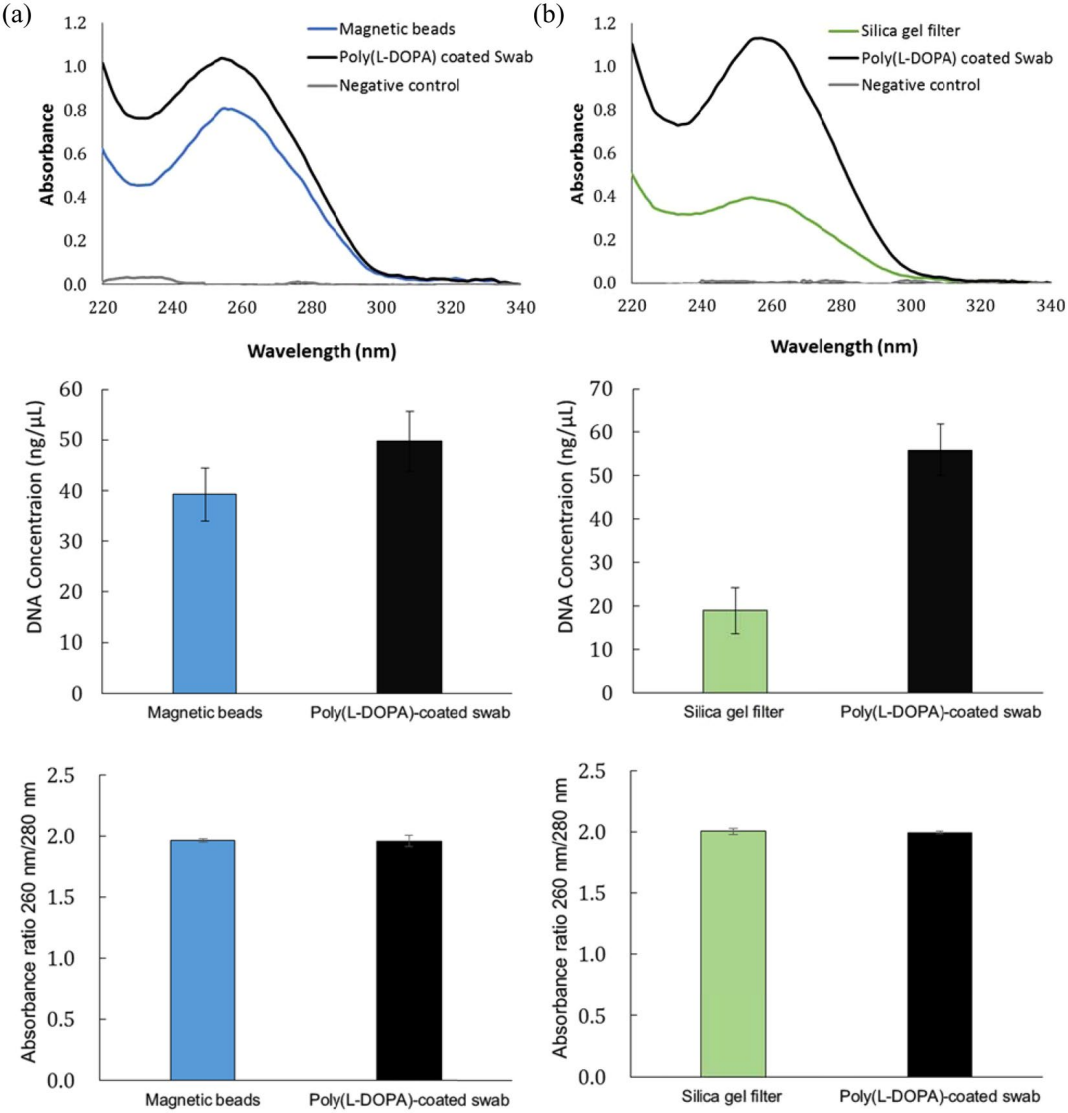
DNA isolation efficiency compared with commercialized kits. UV-vis absorbance spectra of isolated DNA (top), isolated DNA concentration (middle) and its absorbance ratios of 260 nm/280 nm (bottom) compared with (**a**) magnetic bead-based NAI kit (MagListo™ Kit) and (**b**) silica gel filter-based NAI kit (DNeasy Blood & Tissue Kit). Negative control: DNA isolation using poly(L-DOPA)-coated swab from cell brank solution.

DNA isolation from PC-3M cells (1 × 10^6^ cells) carried out with the poly(L-DOPA)-coated swab and magnetic beads according to the MagListo™ 5 M Genomic DNA Extraction Kit protocol showed almost the same appearance in the UV–vis spectra (Fig. 4a). In contrast, DNA isolation from cell blank solution with the swab used as a negative control showed almost no absorption in the UV–vis spectrum. The concentrations of the DNA were 44.7 ng/μL when isolated using the poly(L-DOPA)-coated swab and 39.2 ng/μL when isolated using the magnetic bead. The DNA isolation efficiency of the swab was approximately 14% higher than that of the magnetic beads. The absorbance ratios of 260 nm/280 nm were 1.96 for the poly(L-DOPA)-coated swab and 1.95 for the magnetic beads, with both demonstrating that the isolated DNA were of high purity. In the same manner, DNA isolated using the poly(L-DOPA)-coated swab and that using the silica gel filter according to the DNeasy Blood & Tissue Kit protocol showed identical patterns in the UV–vis spectra (Fig. 4b). Also, DNA isolation from cell blank solution with the swab used as a negative control showed no absorption in the UV–vis spectrum. The concentrations of the isolated DNA were 55.9 ng/μL for the poly(L-DOPA)-coated swab and 19.0 ng/μL for the silica gel filter. This shows that the DNA isolation efficiency of the swab was approximately 3 times higher than that of the silica gel filter. The absorbance ratios of 260 nm/280 nm were 1.99 for the poly(L-DOPA)-coated swab and 2.00 for the silica gel filter, with both demonstrating that the isolated DNA were of high purity.

### DNA isolation after swab-based collection of saliva

**Figure 5:**
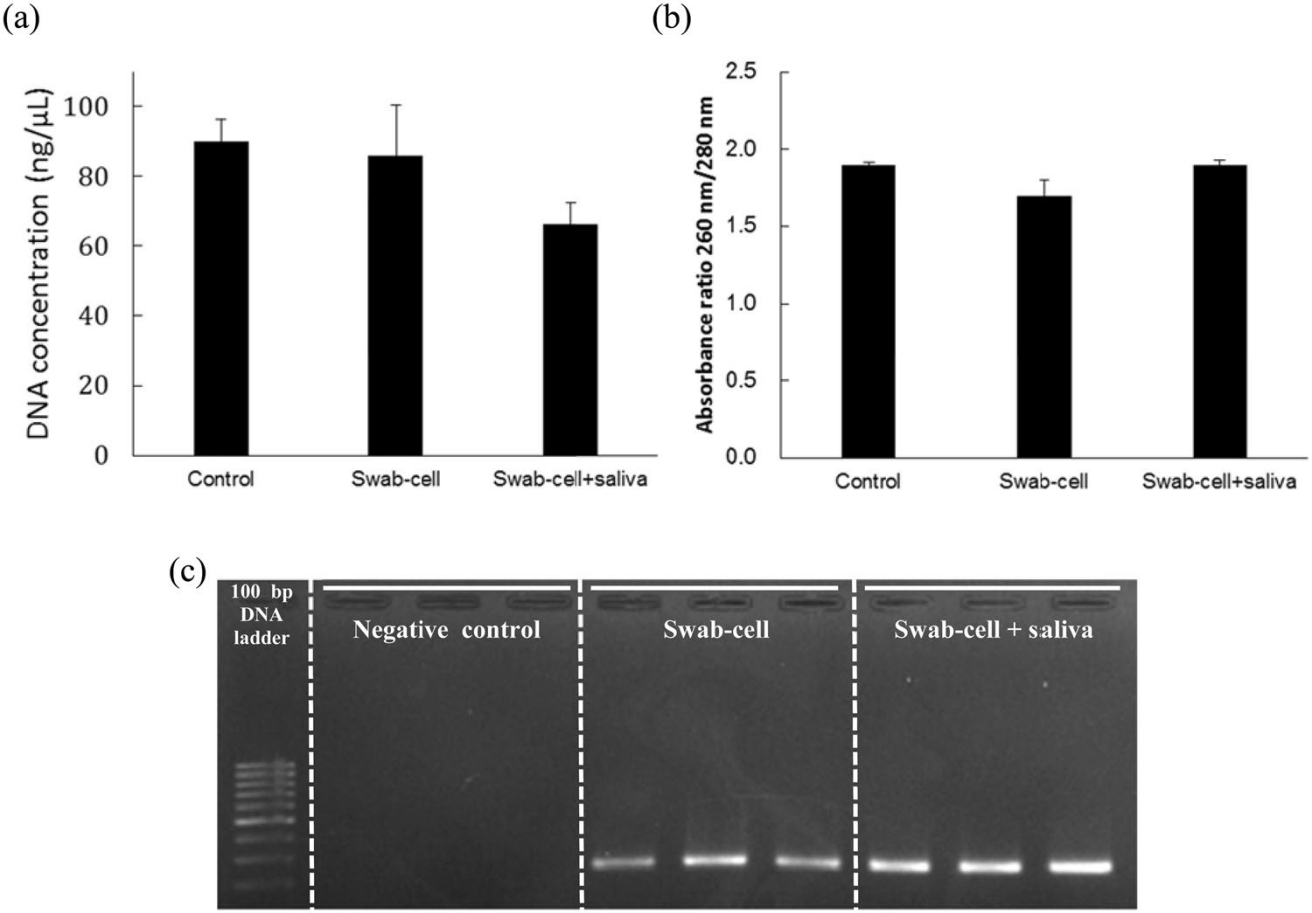
DNA isolation after collecting sample using poly(L-DOPA)-coated swab. (**a**) Isolated DNA concentrations, (**b**) its absorbance ratios of 260 nm/280 nm and (**c**) agarose gel electrophoresis image of GAPDH PCR product using the isolated DNA from cells directly added to lysis buffer (Control), from cells collected by the swab (Swab-cell) and from saliva spiked with cells collected by the swab (Swab-cell + saliva).

In clinical diagnostics, swabs are mainly used to collect the samples by scrapping, such as nasopharyngeal swab, cervical-vaginal swab and fecal swab. We carried out swab-based sample collection and DNA isolation with the poly(L-DOPA)-coated swab using cells (swab-cell) and saliva spiked with cells (swab-cell + saliva). DNA isolation with the swab from cells directly added into lysis buffer was also carried out as a control. As shown in the Fig. 5a, the isolated DNA concentration was 85.9 ng/μL in the case of the swab-cell and 66.4 ng/μL in the case of the swab-cell + saliva, while 90.1 ng/μL in the control. The absorbance ratios of 260 nm/280 nm were between 1.7–1.9 for all samples demonstrating that the isolated DNA were of high purity (Fig. 5b). The DNA isolation from the cell-swab showed similar efficiency as the control. This means that the swab-based cell collection does not affect the DNA isolation efficiency. On the other hand, the DNA isolation from the swab-cell + saliva showed 26% lower efficiency than that of the control. This means that the substances in saliva interfere with swab-based cell collection. However, the isolated DNA from the swab-cell + saliva was successfully amplified by PCR (Fig. 5c). We could confirm the possibility of the poly(L-DOPA)-coated swab for swab-based sample collection and DNA isolation of clinical samples.

### DNA isolation after sterilization

**Figure 6:**
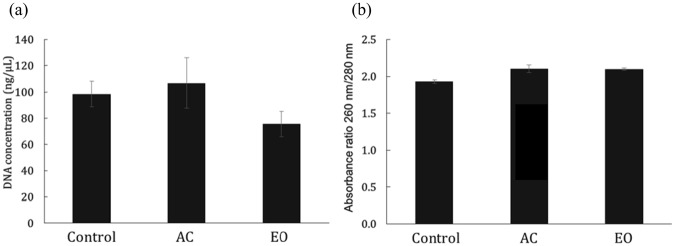
DNA isolation after sterilization of poly(L-DOPA)-coated swab. (**a**) Isolated DNA concentration and (**b**) its absorbance ratios of 260 nm/280 nm using non-sterilized (Control), autoclaved (AC) and ethylene oxide gas (EO) sterilized poly(L-DOPA)-coated swab.

Poly(L-DOPA)-coated swabs must be used after sterilization to directly contact the human body for sample collection. We evaluated the DNA isolation efficiencies after sterilization of the poly(L-DOPA)-coated swab. The poly(L-DOPA)-coated swab was sterilized by autoclaving or by using ethylene oxide (EO) gas. When sterilized by autoclaving (temperature 121 °C, pressure 15 psi, time 15 min), the poly(L-DOPA) coating and the nylon material (melting point 268.7 °C) of the swab did not change, but the plastic material, which supports the swab and has poor heat conductivity, decreased in size. When sterilized with EO gas, no apparent deformation was observed in the poly(L-DOPA)-coated swab. The concentration of the isolated DNA from PC-3M cells (2 × 10^6^ cells) using non-sterilized poly(L-DOPA)-coated swab (control), autoclaved swab (AC), and EO-sterilized swab (EO) were 98.4 ng/μL, 106.8 ng/μL, and 75.6 ng/μL, respectively (Fig. 6a). There was no significant difference between the non-sterilized swab and the autoclaved swab, but the EO-sterilized swab showed that the DNA isolation efficiency was reduced by 24%. Although the alkylation by EO gas may affect the poly(L-DOPA) coating, DNA isolation was possible after sterilization with EO gas. The absorbance ratios of 260 nm/280 nm were 1.93 using control, 2.10 using AC, and 2.10 using EO, with all demonstrating that the DNA isolated were of high purity (Fig. 6b). The sterilized poly(L-DOPA)-coated swab can be applied to the direct collection of human specimens such as nasopharyngeal swabs and cervical-vaginal swabs.

### Fabrication and efficiency test of integrated DNA isolation device

**Figure 7:**
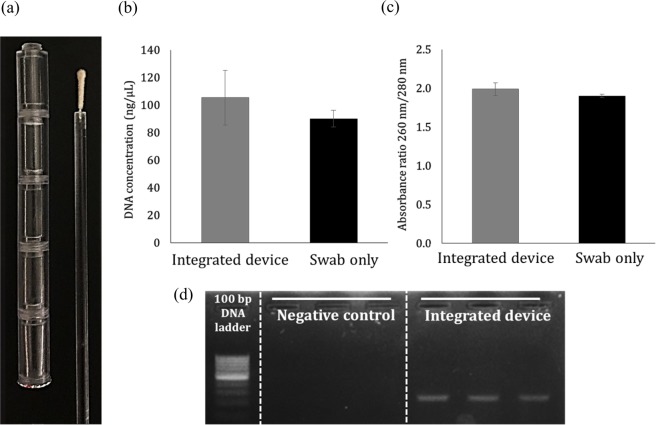
DNA isolation using integrated DNA isolation device. (**a**) Photograph of the actual fabricated device, (**b**) isolated DNA concentration and (**c**) its absorbance ratios of 260 nm/280 nm using the whole device compared with that using the only modified swab, and (**d**) agarose gel electrophoresis image of GAPDH PCR product using the isolated DNA.

The chambers for each buffer were fabricated through 3D printing. The chambers were separated by a membrane. A stick supporting the swab was also fabricated by 3D printing to match the length of the integrated chambers (Fig. 7a). The diameter of the stick is almost identical to the diameter of the hole in the chamber, and an elastic gel-type membrane separating the chamber can tightly seal the hole while the swab and stick pass through the hole to prevent liquid leakage. In addition, the swab is moved upward so that the already interacted buffer cannot mix with the buffers present in the chamber of the next step. The gel-type membrane was already punched with pipet tip and thin silver foil was attached to block the buffer leakage. Therefore, the swab can pass easily through the membrane with only the force to penetrate the thin silver foil. In this device, the DNA isolation buffers in the DNeasy Blood & Tissue Kit (Qiagen) were used. To evaluate the isolated DNA concentration using the integrated device, the PC-3M cells (2 × 10^6^ cells) were injected into the lysis buffer chamber, and the stick with the poly(L-DOPA)-coated swab was then inserted into the chambers by passing through the membrane and allowed to interact with each buffer. After the processes, the elution solution was collected from the top membrane of the device using a syringe. The concentration of isolated DNA using the device was compared with the manual DNA isolation method using only the poly(L-DOPA)-coated swab. As shown in Fig. 7b, the isolated DNA concentrations were 90.1 ng/μL for the poly(L-DOPA)-coated swab and 105.3 ng/μL for the integrated device. It was confirmed that the DNA isolation ability of the poly(L-DOPA)-coated swab was maintained even in the device. The absorbance ratios of 260 nm/280 nm were 1.99 and 1.90, respectively, with both demonstrating that the DNA isolated were of high purity (Fig. 7c). PCR was performed for cellular GAPDH to determine if molecular diagnostics is possible using the isolated DNA in the integrated device. Electrophoresis after PCR amplification demonstrated that GAPDH was amplified from the isolated DNA using the integrated device. Therefore, it was confirmed that molecular diagnosis is possible using our integrated DNA isolation device.

## Conclusion

In this study, we developed a poly(L-DOPA)-coated swab and integrated chambers containing DNA isolation buffers. The DNA isolation efficiency of the poly(L-DOPA)-coated swab was observed to be similar to or higher than that of the conventional commercial kits of the magnetic bead-based method and the silica gel filter-based method. Furthermore, the DNA isolation ability of the poly(L-DOPA)-coated swab was maintained even after swab-based sample collection and sterilization. Therefore, the poly(L-DOPA)-coated swab can collect specimens directly from the human body and continue the DNA isolation process. The integrated device can isolate the DNA by simply passing the swab through the chambers. This device does not require pipetting and centrifugation, or a magnetic bar for buffer exchange; further, it is portable and allows for simple operation. We confirmed that the isolated DNA using the integrated device could be amplified by PCR. This device can be modified to be compact. In addition, it can be linked to an automated PCR device, isothermal amplification systems, or used for non-amplification detection methods for on-site molecular diagnostics. As a future study, we are developing an on-site molecular diagnostic system by adding a detection chamber behind the elution chamber of this device.

## Methods

### Synthesis of poly(L-DOPA)-coated swab

A 3,4-DL-dihydroxyphenylalanine (L-DOPA) (Sigma Aldrich, USA) solution was prepared at a concentration of 2 mg/mL using Tris-HCl buffer (10 mM, pH 8.5). A nasal cavity swab (FLOQ Swabs™, Copan, USA) was immersed in the L-DOPA solution and stirred at room temperature for 20 h. The swab was washed with deionized water to remove the less adhered polymer particles from the surface and dried at 40 °C in a vacuum oven. After the poly(DOPA)-coated swab was immersed in 5-mM NaIO4 (Sigma Aldrich, USA) overnight, the swab was thoroughly washed with water and neutralized by immersion in 10 mL of 0.1-M NaOH for 2 h. After that, the solution was rinsed with sodium acetate buffer (3 M, pH 5.2), Tris-HCl buffer (10 mM, pH 8.5), and distilled water until the UV–vis absorbance of the washing solution was not detected.

### Genomic DNA isolation using poly(L-DOPA)-coated swab

PC-3M cellular DNA was isolated from different cell numbers (1 × 10^5^, 5 × 10^5^, 1 × 10^6^, 2 × 10^6^) using poly(L-DOPA)-coated swabs. The buffers for lysis, binding, washing, and elution in MagListo™ kit (Bioneer, Korea) were used according to its protocol. All incubations with buffers were carried out at room temperature. The final volume of elution buffer was set to 300 μL, so that the swab could be completely immersed in the buffer. The isolated DNA was quantified using a NanoDrop spectrophotometer (ND-2000, Thermo Scientific, Waltham, MA, USA).

PC-3M cellular DNA was isolated using poly(L-DOPA)-coated swabs depending on the sample volume. The buffers for lysis, binding, washing, and elution in MagListo™ kit (Bioneer, Korea) were used according to its protocol. However, the volume of lysis buffer was adjusted to 200 μL, 400 μL, and 600 μL with a constant cell concentration of 2 × 10^6^ cells/mL) and the volume of the binding buffer was increased proportional to the volume of the lysis buffer. The isolated DNA was quantified using the NanoDrop spectrophotometer.

The concentration of isolated DNA using poly(L-DOPA)-coated swabs was compared with two commercially available kits, MagListo™ Kit (Bioneer, Daejeon, Korea) and DNeasy Blood & Tissue Kit (Qiagen, Germany). The PC-3M cell was used at a number of 1 × 10^6^ cells per test. In comparison with each commercialized kit, buffers, its volumes, and incubation times of each kit were also applied to the poly(L-DOPA)-coated swabs. All steps were performed at room temperature, and the final volume of the elution buffer was set to 300 μL. The isolated DNA was quantitatively compared using the NanoDrop spectrophotometer.

PC-3M cell (2 × 10^6^ cells) was collected by scraping with the poly(L-DOPA)-coated swab and the lysis step was carried out with the cell-caught swab. Also, 100 μL of saliva spiked with PC-3M cell (2 × 10^6^ cells) was collected by scraping with the poly(L-DOPA)-coated swab and the lysis step was carried out with the swab. After lysis step, cellular DNA was isolated in the same manner as the previous method using buffers in DNeasy Blood & Tissue Kit. The isolated DNA was quantified using the NanoDrop spectrophotometer.

We carried out the experiments using the saliva according to the guidelines of Samsung Medical Center after receiving approval for the experimental protocol from the Institutional Review Board of Samsung Medical Center.

### Sterilization of poly(L-DOPA)-coated swab

The modified swab was sterilized by autoclave and ethylene oxide (EO) gas sterilization. Autoclaving was carried out at 121 °C, 15 psi for 30 min using a steam sterilizer (HS-5020, Hanshin medical Co., Korea). The EO gas sterilization proceeded at 38 °C for 4.5 h using an EO gas sterilizer (HG-3041 E.P, Hanshin medical Co., Korea). The sterilized swab was used to isolate the cellular DNA using PC-3M (2 × 10^6^ cells/test) in the same manner as the previous method using buffers in DNeasy Blood & Tissue Kit.

### Device fabrication and operation

The integrated DNA isolation device was designed using computer-aided design software, Autodesk Fusion 360. This device was fabricated using 3D printing using polycarbonate, a transparent material. In this device, the DNA isolation buffers of the DNeasy Blood & Tissue Kit were added to each chamber. And then, the chamber was sealed with a membrane to prevent the mixing of reagents. The membrane consisted of a gel-type membrane and a thin silver foil. The gel-type membrane made of hydrocolloid components was already punched with pipet tip and attached with the silver foil. However, the membrane between chambers for lysis and binding consisted only of silver foil to allow the buffers to be mixed in the chambers after passing of the swab. Then, the chambers were assembled step-by-step (lysis chamber, binding chamber, two of washing chamber, and elusion chamber) by screw manner (Figure S1). This stick was fabricated by 3D printing using polycarbonate, which had a hole in the top to hold the swab.

The PC-3M 2 × 10^6^ cells were added to the lysis chamber. Then, the cellular DNA was isolated by passing the poly(L-DOPA)-coated swab through the membrane. The swab was positioned upright. After passing through the membrane, the swab was allowed to interact with the buffer in the chamber with gentle vortexing. The isolated DNA was collected from the final membrane using a syringe and quantified using the NanoDrop spectrophotometer.

### Polymerase Chain Reaction

Polymerase chain reaction (PCR) was performed against the cellular DNA isolated from PC-3M using the poly(L-DOPA)-coated swab and the integrated DNA isolation device. The forward and reverse primer sequences for human glyceraldehyde‐3‐phosphate dehydrogenase (GAPDH) were synthesized as 5′-GAAGGTGAAGGTCGGAGT-3′ and 5′-AAGATTGGTGATGGGATTTC-3′, respectively^17^. We added 3.8 μL of elution solution containing DNA isolated from PC-3M, primers (final concentration, 0.2 μM), and 2X Taq premix Ver.1.1 (BAT113-M005, Bioassay, Korea). The PCR amplification conditions were as follows: 95 °C pre-denaturation step for 5 min, denaturation step at 95 °C for 10 s, annealing step at 60 °C for 20 s, and elongation step at 72 °C for 10 s (30 cycles). PCR amplification was performed using a 96-well thermal cycler (Applied Biosystems, USA). The amplified PCR products were identified by electrophoresis using a 2% agarose gel.

## Supplementary information

Development of a DNA isolation device using poly(3,4-dihydroxy-L-phenylalanine)-coated swab for on-site molecular diagnostics
