# Supplementary material for: Development of a DNA isolation device using poly(3,4-dihydroxy-L-phenylalanine)-coated swab for on-site molecular diagnostics
Source: Sci Rep. 2019 May 31;9:8144. doi: 10.1038/s41598-019-44527-2 (PMC6544620; doi:10.1038/s41598-019-44527-2)
Supplement: Supplementary file 1 — Development of a DNA isolation device using poly(3,4-dihydroxy-L-phenylalanine)-coated swab for on-site molecular diagnostics [file 41598_2019_44527_MOESM1_ESM.docx]

Supplementary Information

**Development of a DNA isolation device using poly(3,4-dihydroxy-L-phenylalanine)-coated swab for on-site molecular diagnostics**

Hyun-Ju Park,^a†^ Heesoo Cho,^b†^ Ho Sang Jung,^c^ Baek Hwan Cho,^a,b^* Min-Young Lee^a,b^*

*
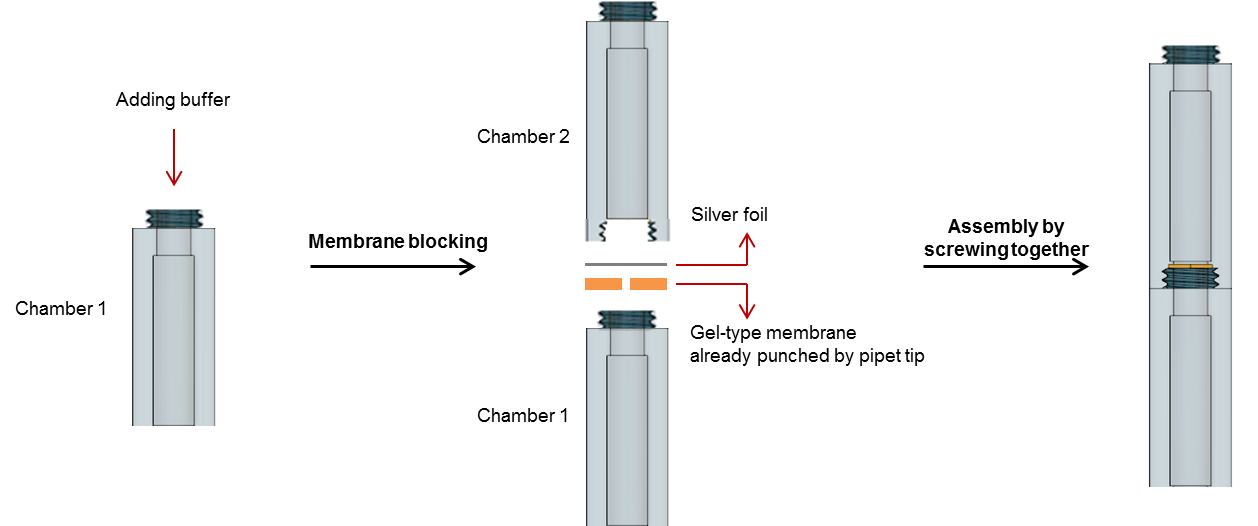
*

**Figure S1. Assembly processes of chambers for integrated DNA isolation device.**
